# Influence of Water Solute Exposure on the Chemical Evolution and Rheological Properties of Asphalt

**DOI:** 10.3390/ma11060983

**Published:** 2018-06-11

**Authors:** Ling Pang, Xuemei Zhang, Shaopeng Wu, Yong Ye, Yuanyuan Li

**Affiliations:** State Key Laboratory of Silicate Materials for Architectures, Wuhan University of Technology, Wuhan 430070, China; lingpang@whut.edu.cn (L.P.); zhangxuemei@whut.edu.cn (X.Z.); wusp@whut.edu.cn (S.W.); liyuanyuan@whut.edu.cn (Y.L.)

**Keywords:** asphalt, water solute exposure, aqueous solute compositions, chemical evolutions, rheological properties

## Abstract

The properties of asphalt pavement are damaged under the effects of moisture. The pH value and salt concentration of water are the key factors that affect the chemical and rheological properties of asphalt during moisture damage. Four kinds of water solutions, including distilled water, an acidic solution, alkaline solution and saline solution were used to investigate the effects of aqueous solute compositions on the chemical and rheological properties of asphalt. Thin-layer chromatography with flame ionization detection (TLC-FID), Fourier transform infrared (FTIR) spectroscopy and dynamic shear rheometer (DSR) were applied to investigate the components, chemistry and rheology characteristics of asphalt specimens before and after water solute exposure. The experimental results show that moisture damage of asphalt is not only associated with an oxidation process between asphalt with oxygen, but it is also highly dependent on some compounds of asphalt dissolving and being removed in the water solutions. In detail, after immersion in water solute, the fraction of saturates, aromatics and resins in asphalt binders decreased, while asphaltenes increased; an increase in the carbonyl and sulphoxide indices, and a decrease in the butadiene index were also found from the FTIR analyzer test. The rheological properties of asphalt are sensitive to water solute immersing. The addition of aqueous solutes causes more serious moisture damage on asphalt binders, with the pH11 solution presenting as the most destructive during water solute exposure.

## 1. Introduction

Asphalt pavement, for its excellent driving performance and low noise advantages, is widely applied in the world [1]. During the service phase of asphalt pavement, moisture from the natural environment can diffuse into the asphalt binder, which results in the attenuation of the pavement performance of the asphalt mixture [2,3]. Recent studies suggest that the interaction between water oxygen molecules and asphalt can cause oxidation and aging of asphalt and increase its stiffness and viscosity [4,5]. Also the water may dissolve part of the components of asphalt, which softens the asphalt, reduces the adhesion between the asphalt binder and aggregate, and reduces the cohesion capability inside the asphalt binder. As a result, the mechanical properties of asphalt concrete and the binder are degraded [6,7,8,9].

Moisture damage is considered one of the most important factors affecting asphalt mixture durability [10,11,12,13]. Water is closely related to the moisture damage of asphalt pavement during its service phase, and the different aqueous solute compositions of water, such as in areas of acid rain, seaboards, or saline and alkaline land, may cause different moisture damage effects on an asphalt binder [14,15]. In salty and humid environments, the strength of asphalt mixture deteriorates easily because of moisture damage from water infiltration and salt chemical corrosion [16,17]. Following immersion in acid rain solutions, the pavement performance of the asphalt mixtures decreased with the decrease in the solution pH value [18]. In an acid rain area, where rainfall with pH < 3 occurs [19], and in saline and alkaline land areas where the pH value of soil may be over pH10 [20], the pavement is subjected to around 10% sodium chloride corrosion damage under snow melting [21]. The reduction in the properties of the asphalt mixture has been related to component changes under the effect of aqueous solute in the water solution. An important consideration for understanding the asphalt moisture damage process in different water solutions is that the chemical composition and rheological properties of asphalt may not be the same as at the asphalt-water surface where environmental factors have the greatest impact. Thus, understanding the process of moisture damage in asphalt under environmental exposure, especially the role and effect of the aqueous solute component in this process, is very important to the research and prevention of moisture damage to asphalt pavement.

However, there is little research on the effects of aqueous solute composition on the chemical and rheological properties of asphalt. Besides, due to the test conditions and material differences, the degree of influence of different aqueous solute composition factors on the chemical and rheological properties of asphalt is unclear. The main objective of this paper is to analyze the evolution of the chemical and rheological properties of asphalt at different pH values of water solution and saline solution conditions, hence, four kinds of water solution, including distilled water, acidic, alkaline and saline solution, were used to immerse the asphalt. The effects of water solute exposure on asphalt chemical composition, construction and rheology were investigated using four compositions which were analyzed using thin-layer chromatography with flame ionization detection (TLC-FID), Fourier transform infrared spectroscopy (FTIR) and dynamic shear rheometer (DSR).

## 2. Materials and Experimental Methods

### 2.1. Materials

The 70^#^ asphalt and SBS modified asphalt were obtained from Hubei Guochuang Hi-tech Material Co., Ltd., Wuhan, China. Their basic properties are exhibited in Table 1. The ductility test temperatures of 70^#^ asphalt and SBS modified asphalt were 10 °C and 5 °C, respectively.

### 2.2. Preparation of Solutions

In different regions, the aqueous solute composition of water solutions are different in nature. For example, the pH value of rainfall may be around 3 in acid rain areas, the pavement is subjected to around 10% sodium chloride corrosion damage under snow melting, or the pH value of the solution may be over pH10 in saline and alkaline land. So, pH3 acid solution, 10% NaCl salt solution and pH11 alkali solution were selected in this paper. The preparation methods of these are described below. 

Artificial acid solution was prepared to simulate the natural ingredients in acid rain, such as the SO_4_^2−^ and NO_3_^−^ anions. It was prepared with excellent pure sulfuric acid and nitric acid by the serial dilution method [22], the molar ratio of sulfuric acid and nitric acid was 9:1, the pH value was 3. Alkaline solution was prepared with sodium hydroxide, and the pH value was 11. The water solute immersing tests of asphalt binders were conducted at 25 °C and normal pressure, the pH of the solution was measured with a precise pH test paper. Sodium chloride was diluted with distilled water, the concentration of which was 10%.

### 2.3. Water Solute Immersing Tests

First, a circular glass dish, with a diameter of 100 mm and height of 18 mm, was washed with deionized (DI) water. The selected 6 g of each asphalt binder was poured onto the glass dish and put into an oven at a constant temperature of 120 °C for 10 min to form a smooth asphalt film; the thickness of asphalt film was about 0.76 mm. After that, the samples were taken out and cooled to room temperature.

Then, 40 mL of the solutions were poured into the dish and immersed the asphalt films, the glass dish was capped. The temperature of the immersing water solute was 25 °C, the test times for 70^#^ asphalt were designated as 7 days and 14 days, and 14 days and 28 days for the SBS modified asphalt. During the water solute immersion test, the solutions were replaced every week because the carboxylic acid of asphalt can be dissolved and reduce the pH value of the solution [23]. After the water solute immersion test, in order to make sure the water was fully evaporated from the surface of the asphalt samples, all the asphalt specimens were put into an oven at a constant temperature of 150 °C for 30 min. Subsequently, the asphalt specimens were separated from the glass dish with a spatula.

### 2.4. Characterization Methods

#### 2.4.1. Experimental Program Plan

**Figure 1:**
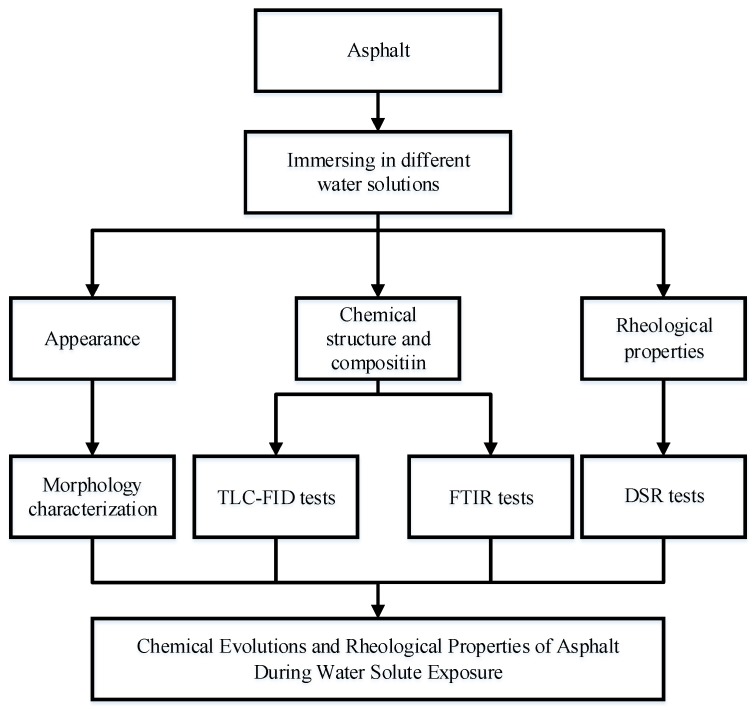
Experimental program plan.

The experimental program plan is shown in Figure 1. At the first, two kinds of asphalt (70^#^ asphalt and SBS modified asphalt) were prepared to be immersed in different water solutions (distilled water, acid solution, alkaline solution and sodium chloride solution). Secondly, the TLC-FID tests, FTIR tests and DSR tests were used to characterize the effect of immersion in water solute on the chemical evolution and rheological properties of asphalt. To increase the accuracy, two times replicate tests were carried out. Lastly, the mechanism of asphalt during moisture damage was summarized.

#### 2.4.2. Four Compositions Analysis

Asphalt consists of various molecular weights of hydrocarbons and its derivatives and based on the relative molecular size and polarity of asphalt, it can be divided into four components, namely saturates, aromatics, resins and asphaltenes [24,25]. In order to test the impact of aqueous solute composition on these four components of asphalt, TLC-FID (Iatron Laboratories Inc., Tokyo, Japan) was used to analyze the four components of asphalt before and after water solute exposure. Two percent (*w*/*v*) solutions of asphalt binders were prepared in dichloromethane, and 1 μL sample solution was spotted on chromarods. There was a three-stage process for the separation of asphalt fractions. The first stage was in n-heptane (70 mL) and expanded to 100 mm of the chromarods, the second stage in toluene/n-heptane (70 mL, 4/1 by volume) was developed to 50 mm of the chromarods, and the last development was in toluene/ethanol (70 mL, 11/9 by volume) and expanded to 25 mm of the chromarods. The solvent was dried in an oven at 80 °C after each stage. Then, the chromarods were scanned in the TLC-FID analyzer. Four chromarods were tested for each sample, and finally the average values were used as the results.

#### 2.4.3. Fourier Transform Infrared (FTIR)

A Thermo Nicolet Nexus FTIR spectrophotometer (Thermo Fisher Scientific, Waltham, MA, USA) was used to test the chemical structure of asphalt binders before and after water solute exposure. The asphalt carbon disulfide solutions were prepared with a concentration of 5 wt % asphalt binders. All samples were obtained using a 0.1 mm path length KBr cell. Spectra were recorded using the following settings: number of scans 64; gain 1; apodization weak; and resolution 4. The change in chemical structure due to asphalt oxidation aging could be obtained by the calculation of functional and structural indices of some groups from the FTIR spectra. The oxidation aging of asphalt increases the area of carbonyl and the sulphoxide absorption peak, while greatly decreasing the area of butadiene double bonds absorption peak, the area of these absorption peaks are closely related with the degree of aging of asphalt, thus, carbonyl group C=O (centered around 1700 cm^−1^), sulphoxide group S=O (centered around 1030 cm^−1^) and chain segments of butadiene C=C (centered around 968 cm^−1^) could be used to characterize the degree of aging of the asphalt [26,27,28]. The carbonyl index (*I_C=O_*), sulphoxide index (*I_S=O_*) and butadiene index (*I_SBS_*) can be calculated according to Equations (1)–(3).

(1) $$I_{C=O}=\frac{\mathrm{Area}\;\mathrm{of}\;\mathrm{the}\;\mathrm{carbonyl}\;\mathrm{band}\;\mathrm{centered}\;\mathrm{around}1{700\;\mathrm{cm}}^{-1}}{\sum \mathrm{Area}\;\mathrm{of}\;\mathrm{the}\;\mathrm{spectral}\;\mathrm{bands}\;\mathrm{between}2000\;\mathrm{and}\;600{\;\mathrm{cm}}^{-1}}$$

(2) $$I_{S=O}=\frac{\mathrm{Area}\;\mathrm{of}\;\mathrm{the}\;\mathrm{sulphoxide}\;\mathrm{band}\;\mathrm{centered}\;\mathrm{around}1{030\;\mathrm{cm}}^{-1}}{\sum \mathrm{Area}\;\mathrm{of}\;\mathrm{the}\;\mathrm{spectral}\;\mathrm{bands}\;\mathrm{between}2000\;\mathrm{and}\;600{\;\mathrm{cm}}^{-1}}$$

(3) $$I_{SBS}=\frac{\mathrm{Area}\;\mathrm{of}\;\mathrm{the}\;\mathrm{butadine}\;\mathrm{band}\;\mathrm{centered}\;\mathrm{around}\;968\;{\mathrm{cm}}^{-1}}{\sum \mathrm{Area}\;\mathrm{of}\;\mathrm{the}\;\mathrm{spectral}\;\mathrm{bands}\;\mathrm{between}2000\;\mathrm{and}\;600{\;\mathrm{cm}}^{-1}}$$

#### 2.4.4. Dynamic Shear Rheometer (DSR) Test

The dynamic rheological properties of asphalt were investigated with a dynamic shear rheometer (MCR101, Anton Paar company, Graz, Austria) under a parallel plate configuration. A temperature sweep test from −10 °C to 30 °C with an increment of 2 °C/min was performed under a strain-controlled mode, the constant frequency was 10 rad/s. The specifications followed AASHTO T 315. The strain sweep test was carried out at −10 °C to determine whether 0.1% strain lies within the linear viscoelastic range of the aged binder in advance. The strain was maintained at 0.1% so that all testing would lie within the linear viscoelastic range. Moreover, the diameter of the plate was 8 mm, and the gap between the plates was 2 mm.

## 3. Results and Discussion

### 3.1. Water Solutions’ Effect on the Appearance of Asphalt

**Figure 2:**
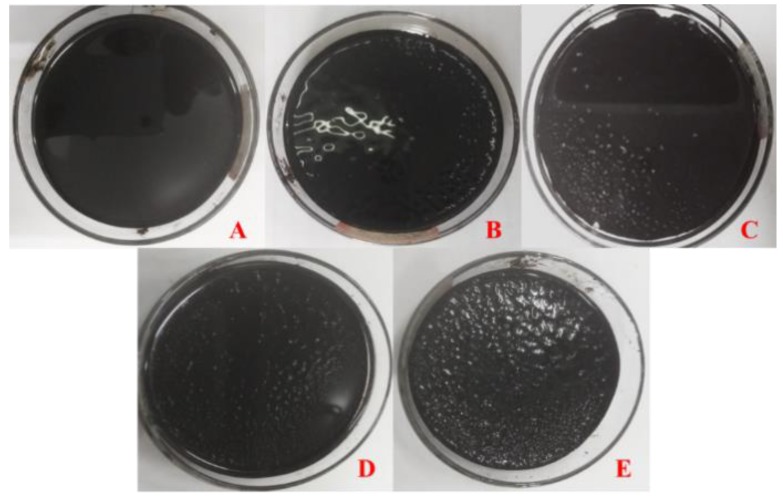
70^#^ asphalt appearance before and after 7 days water solution immersion (**A**) original sample; (**B**) distilled water; (**C**) 10% NaCl salt solution; (**D**) pH3 acid solution; (**E**) pH11 alkali solution.

Figure 2 displays the appearance of 70^#^ asphalt after 7 days of the water solute immersion test. As can be seen, the surface exhibits obvious differences after immersion in different water solutions. Before water solute immersion, the surface of 70^#^ asphalt is fairly even, however, after immersion in the four types of water solutions, the asphalt surface tends to be rougher and some micro bumps or pits can be observed. In detail, the roughness increases in the following order, pH11 alkaline solution > pH3 acidic solution > 10% NaCl saline solution > distilled water. The uneven surfaces may be caused by etching of the water solutions, that is, they are the results of moisture damage, also, the moisture damage effect of the pH11 alkaline solution immersion was the most serious.

**Figure 3:**
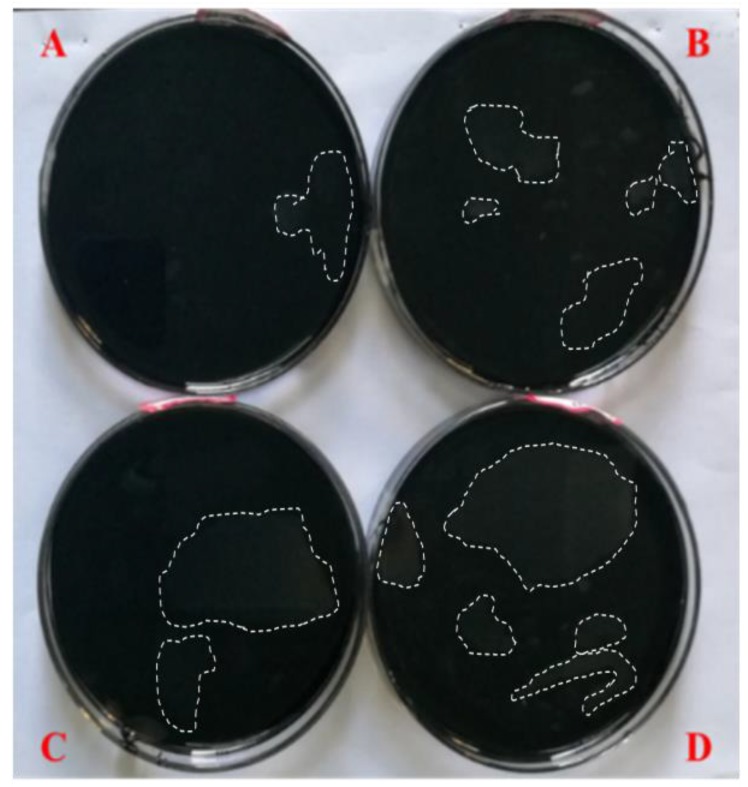
Water solutions’ appearance after 7 days immersion (**A**) distilled water; (**B**) 10% NaCl salt solution; (**C**) pH3 acid solution; (**D**) pH11 alkali solution.

**Figure 4:**
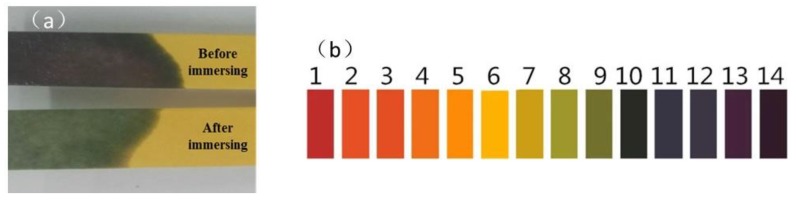
(**a**) The pH value change of alkaline solution. (**b**) The pH paper color swatches after 7 days immersing asphalt.

Figure 3 shows the water solutions after 7 days of the water solute immersion test. It can be found from Figure 3 that some light-yellow oil patches exist on the surfaces of the four water solutions (marked with a white dashed line). The oil patches may derive from insoluble compounds of asphalt under the action of hydrostatic pressure, which are pushed off the asphalt surface and float on top of the water solutions. The changes in the features of the water solutions are consistent with the surface roughness of asphalt after being immersed in water solute, which indicates that the water solutions can dissolve and remove some compounds of asphalt and result in the damage of asphalt. In addition, the order of the oil patch size is pH11 alkaline solution > pH3 acidic solution > 10% NaCl saline solution > distilled water; therefore, the degree of damage caused by the pH11 alkaline solution, pH3 acidic solution and 10% NaCl saline solution are higher than that of distilled water. At the same time, the change in pH value of the alkaline solution after immersion for 7 days is shown in Figure 4, the pH value of the alkaline solution is about 11 before immersing the asphalt, however, the pH value is about 9 after immersing the asphalt, which means the alkaline solution is neutralized by some acidic composition of the asphalt, or that acid from the asphalt can be dissolved in the solution and reduce the pH value of alkaline solution.

### 3.2. Four Component Fractions Analysis

The effect of the aqueous solute compositions on the component fractions of asphalt was studied with TLC-FID, the fractions of the four components of 70^#^ asphalt before and after the water solute immersion test are shown in Table 2.

From Table 2, the fraction of asphaltenes increases, while the fractions of saturates, aromatics and resins tend to decline. For example, after 14 days moisture aging, the fraction of asphaltenes increases to 13.01% in distilled water, 14.68% in 10% NaCl saline solution, 18.89% in pH3 acidic solution, and 19.68% in pH11 alkaline solution, compared with 11.31% of original sample; the fraction of resins fluctuates in distilled water, and decreases observably from 34.46% to 32.70% in 10% NaCl saline solution, to 30.49% in pH3 acidic solution, and to 28.11% in pH11 alkaline solution. Similarly, saturates decline from 15.35% to 15.02% in distilled water, to 13.86 in 10% NaCl saline solution, to 12.29% in pH3 acidic solution, and slightly to 13.57% in pH11 alkaline solution as well; and the fraction of aromatics vary in a relatively narrow range from 38.64% to 37.56%. After water solute immersion, especially with the addition of the aqueous solute of acidic, alkaline and saline compositions, the components with relatively low molecular weight, including saturates and resins decrease while the asphaltene component increases. 

The component changes of asphalt binders are caused by the absorption of oxygen, and by the dissolution of some hydrophilic groups and water-soluble substances of asphalt, these two actions occur simultaneously during immersion in water solute. The oxidation can be derived from the air-water-asphalt interaction and the oxygen in the water solutions [4]. Compared to the saturates and asphaltenes, aromatics and resins are more susceptible to oxidation and the polar or ionic chemical groups in the resins can also be solvated by water [23,29,30,31], therefore, there are more asphaltenes and less aromatics and resins after being immersed in water solute. With the water-soluble substances of asphalt solvated and displaced by water, some saturates presumably were seeped up, and pushed by hydrostatic pressure up to the surface of water and constitute a part of oil patch because it is water insoluble and less dense than water, resulting in a reduction of saturates. The decrease in the saturates of asphalt exposed to water solute was a little different compared to common oxidized aging in heat-oxidized and UV-oxidized aging. Thus, the oxidation of asphalt and the effect of dissolution and removal of some compounds of asphalt in water solutions seems to occur simultaneously, which changes the content and proportion of each component in asphalt. In addition, with an increase of water solute immersion time, the change in amplitude of the components of asphalt binders increases.

The colloidal structure of asphalt can be represented by the Gaestel index (*Ic*), and the *Ic* value is calculated according to the Equation (4) [28]. The increase in *Ic* is used to be characteristic of asphalt heat-oxidized and UV-oxidized aging usually, therefore the *Ic* value is also used to investigate the colloidal structure change of asphalt before and after moisture aging.

(4) $$I_{C}=\frac{\mathrm{aromatics}+\mathrm{resins}}{\mathrm{saturates}+\mathrm{asphaltenes}}$$

**Figure 5:**
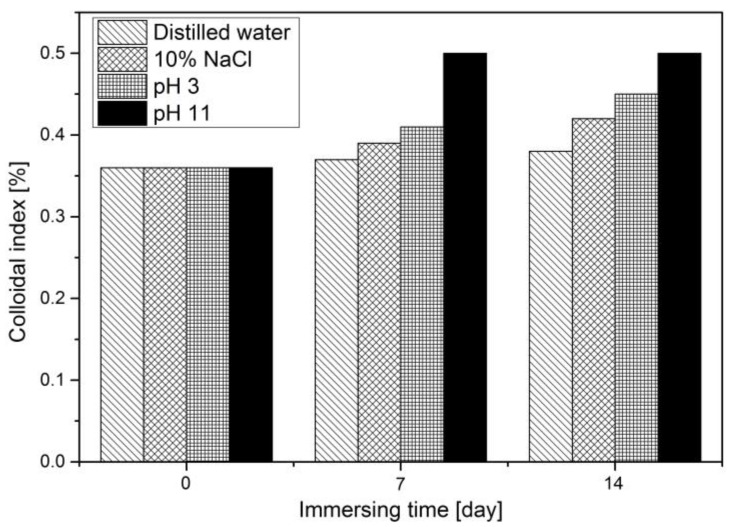
Gaestel index of the 70^#^ asphalt before and after water solute immersion.

The results are shown in Figure 5. As shown in Figure 5, the Gaestel index values of 70^#^ asphalt increase over 7 days and 14 days water solute immersing, which means more gel-like asphalt has formed after water solute exposure, especially after immersion in pH11 alkaline solution. 

### 3.3. Chemical Structure Analysis

**Figure 6:**
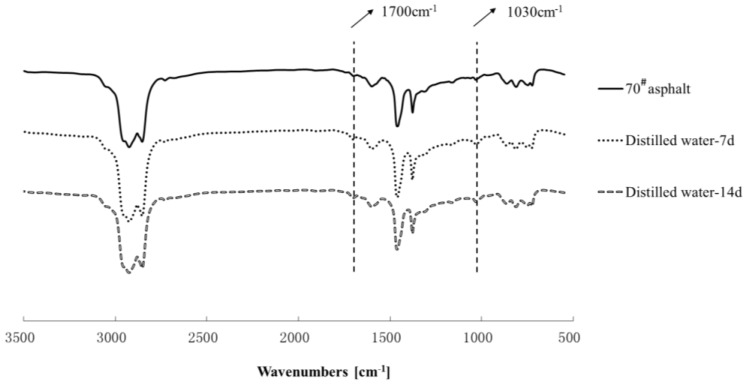
FTIR spectra of the 70^#^ asphalt before and after distilled water aging.

**Figure 7:**
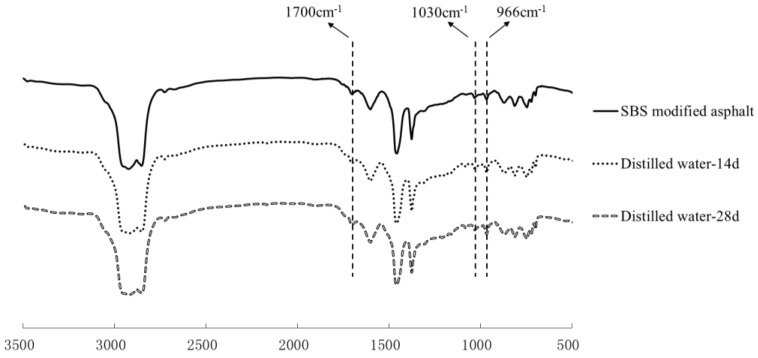
FTIR spectra of the SBS modified asphalt before and after distilled water aging.

The chemical structures of asphalt binders were tested by the FTIR. The results of the FTIR spectrums of 70^#^ asphalts and SBS modified asphalt are shown in Figure 6 and Figure 7, respectively. Compared to the spectra of the origin asphalt, after immersing in water solute, the carbonyl and sulphoxide absorption peaks tend to be more obvious, while the butadiene absorption bands decrease obviously, which shows that more carbonyl and sulphoxide groups were generated, and the chain segments of butadiene decomposed due to immersing in distilled water.

The results of group indices of 70^#^ asphalt and SBS modified asphalt before and after immersion in different water solutions are shown in Table 3.

From Table 3, both the carbonyl index and the sulphoxide index of asphalt increase continuously with extended solution immersion time, which shows the interaction between the water and asphalt can cause the oxidation of asphalt and form more asphaltene components. In addition, both the carbonyl index and sulphoxide index are the highest after immersion in pH11 alkaline solution, followed by the pH3 acid solution, 10% NaCl saline solution and distilled water, therefore, saline, alkaline and acidic components can increase the damage rate of asphalt binders during water solute immersion. The increase of the carbonyl and sulphoxide index were relatively faster when the immersion time is less than 7 days, however, there is a decrease in this trend when the immersion time is from 7 days to 14 days. It can be found from Table 4 that both the carbonyl index and sulphoxide index of SBS modified bitumen experience a similar change tendency as 70^#^ asphalt. With respect to the butadiene index, it was found to reduce after moisture aging and decrease continuously with extended immersion time from 0 days to 28 days, which indicates that the chain segments of butadiene in SBS experience significant degradation.

The results of FTIR spectra demonstrate that oxidation of asphalt and degradation of SBS modifier occur simultaneously during water solute immersion. The aqueous solute in the solutions, that is, the saline, alkaline and acidic components promote the oxidation of asphalt, and the pH11 alkaline solution has the most severe effect during water solute immersion.

### 3.4. Rheological Properties Analysis

**Figure 8:**
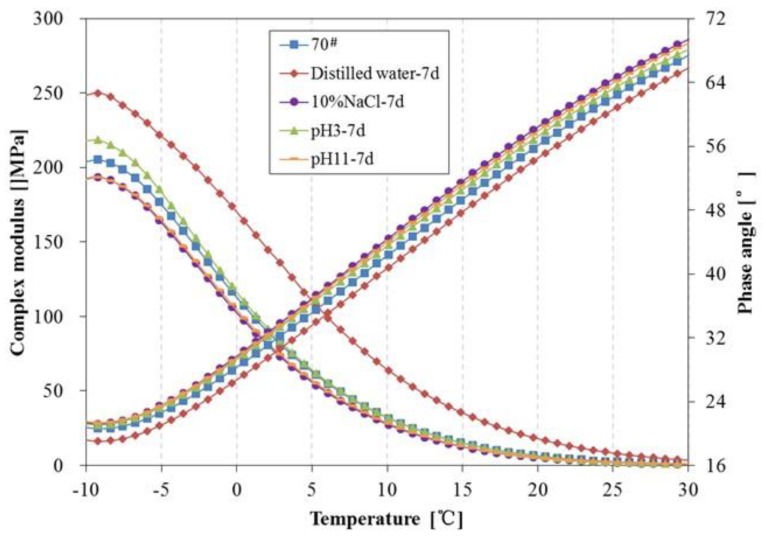
Complex modulus and phase angle of 70^#^ asphalt before and after 7 days immersion.

**Figure 9:**
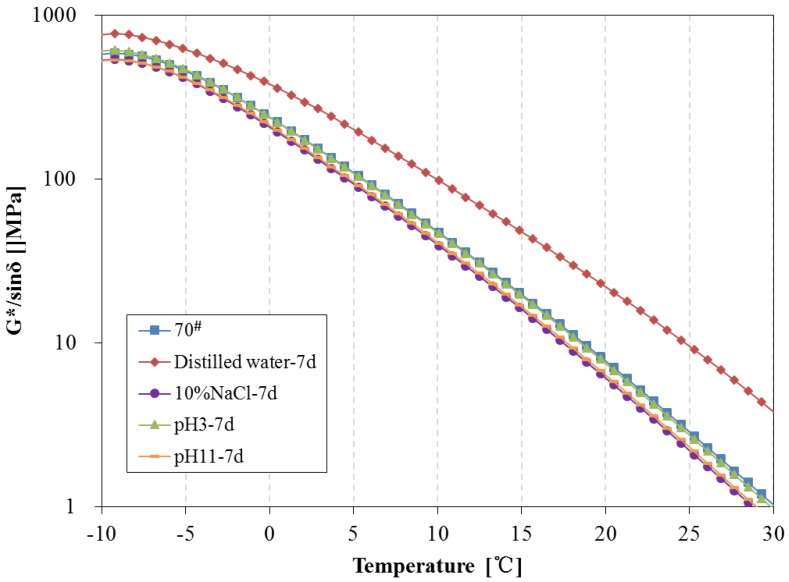
Rutting parameter of 70^#^ asphalt before and after 7 days immersion.

**Figure 10:**
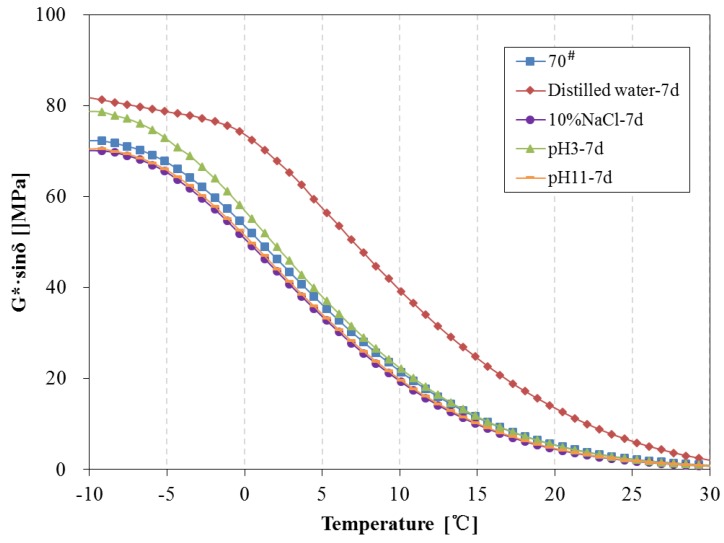
Fatigue factor of 70^#^ asphalt before and after 7 days immersion.

The above tests show there are some changes in chemical composition and structure can be observed during water solute immersing; the newly-formed material structure may cause certain effects on the rheological properties of asphalt. In this paper, the DSR was used to investigate the rheological properties of asphalt binders before and after being immersed in water solute. The complex modulus, phase angle, rutting parameter (G*/sinδ) and fatigue parameter (G*·sinδ) of 70^#^ asphalt from −10 °C to 30 °C before and after 7 days of water solute immersion are shown in Figure 8, Figure 9 and Figure 10.

From Figure 8, after 7 days immersion, the complex modulus and phase angle of 70^#^ asphalt binders changed significantly. The complex modulus of 70^#^ asphalt immersed in distilled water is higher than that of the original 70^#^ asphalt, and the complex modulus of asphalt immersed in the pH11 alkaline solution and 10% NaCl saline solution are lower, while there is almost no difference between the complex modulus of asphalt binders immersed in the pH3 acidic solution and original asphalt. The phase angle of 70^#^ asphalt immersed in distilled water is lower than that of the original 70^#^ asphalt, and the phase angle of 70^#^ asphalt immersed in the other three kinds of water solutions are higher. The G*/sinδ of 70^#^ asphalt before and after 7 days immersion is shown in Figure 9, which demonstrates the permanent deformation resistance of asphalt under repeated loads. The rutting parameter of asphalt immersed in distilled water is apparently higher than that of the original asphalt, however, the rutting parameter of asphalt immersed in the pH11 alkaline solution and 10% NaCl saline solution are lower, and the rutting parameter of asphalt binders immersed in the pH3 acidic solution is close to the original asphalt. From Figure 10, after 7 days immersion, the G*·sinδ of 70^#^ asphalt immersed in distilled water and pH3 acidic solution is apparently higher than that of the original asphalt, while the fatigue factor of asphalt immersed in the pH11 alkaline solution and 10% NaCl saline solution are slightly lower. The lower the G*·sinδ is, the better the fatigue cracking resistance of asphalt binder is. Therefore, distilled water and pH3 acidic solution can significantly decrease the resistant fatigue ability of 70^#^ asphalt, and the effects of 7 days immersion in pH11 alkaline or 10% NaCl saline solution on the resistant fatigue ability of 70^#^ asphalt are not obvious.

The change tendency of 70^#^ asphalt in Figure 8, Figure 9 and Figure 10 show that the addition of aqueous solute changes the variation trend of rheological properties of asphalt in the early phase of immersion. Previous studies showed that some asphalt components forming cohesion capability inside the asphalt are easily solvated and displaced by water [28]. This displacement should weaken the asphalt’s inside bond and decrease the complex modulus, rutting parameter and fatigue factor, while increasing the phase angle. This process may be reinforced when the aqueous solute of acidic, alkaline or salt compositions exists in the water solutions.

**Figure 11:**
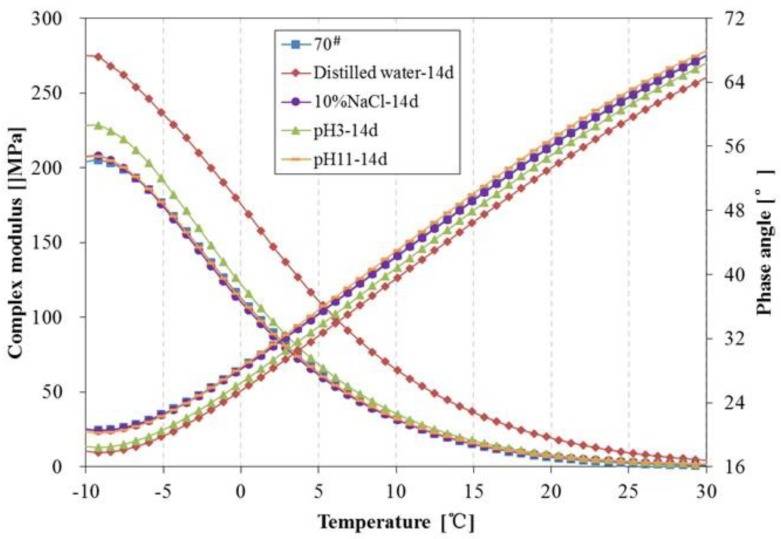
Complex modulus and phase angle of 70^#^ asphalt before and after 14 days immersion.

**Figure 12:**
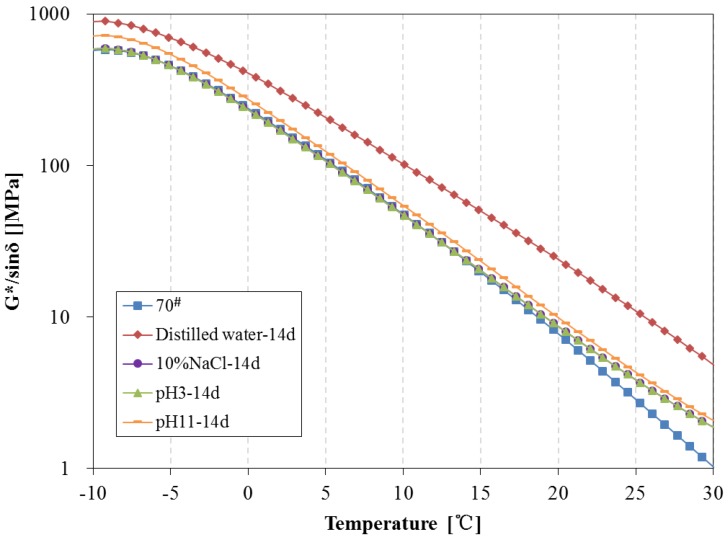
Rutting parameter of 70^#^ asphalt before and after 14 days immersion.

**Figure 13:**
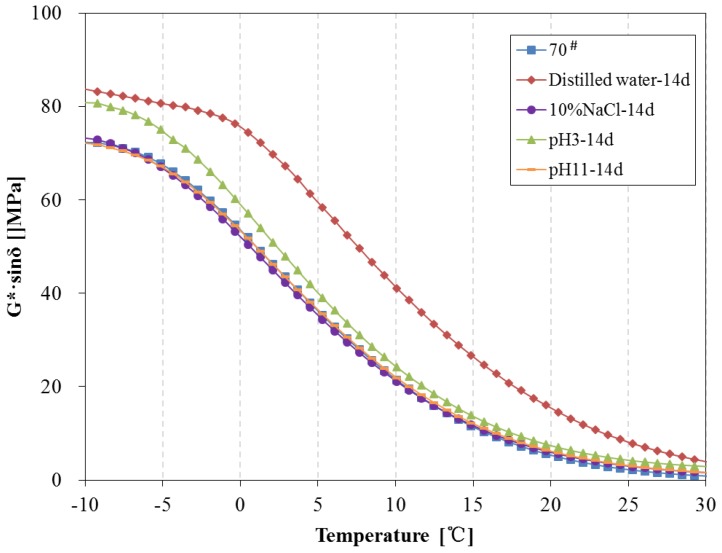
Fatigue factor of 70^#^ asphalt before and after 14 days immersion.

The complex modulus, phase angle, rutting parameter (G*/sinδ) and fatigue parameter (G*·sinδ) of 70^#^ asphalt before and after 14 days of water solute immersion are shown in Figure 11, Figure 12 and Figure 13. From Figure 11, the complex modulus increases and phase angle clearly decreases after 14 days of immersion. From Figure 12, the G*/sinδ of 70^#^ asphalt immersed in the pH11 alkaline solution, 10% NaCl saline solution and pH3 acidic solution are higher than that of the original 70^#^ asphalt. In Figure 13, the G*·sinδ of 70^#^ asphalt immersed in the pH11 alkaline solution and 10% NaCl saline solution are similar to that of the original 70^#^ asphalt, and the G*·sinδ of asphalt immersed in the distilled water and pH3 acidic solution is higher than that of the original 70^#^ asphalt. The effects of distilled water immersion on the rheological performance of 70^#^ asphalt is most significant after 14 days immersion, followed by the pH3 solution on the complex modulus, phase angle and G*·sinδ, and the pH11 alkaline solution on G*/sinδ. Because of the displacement effect of water solutions with aqueous solute, more time is needed to observe the effect of the water solution, thus, the increase in the complex modulus, G*/sinδ and G*·sinδ, and the decrease of phase angle appear after 14 days immersion.

**Figure 14:**
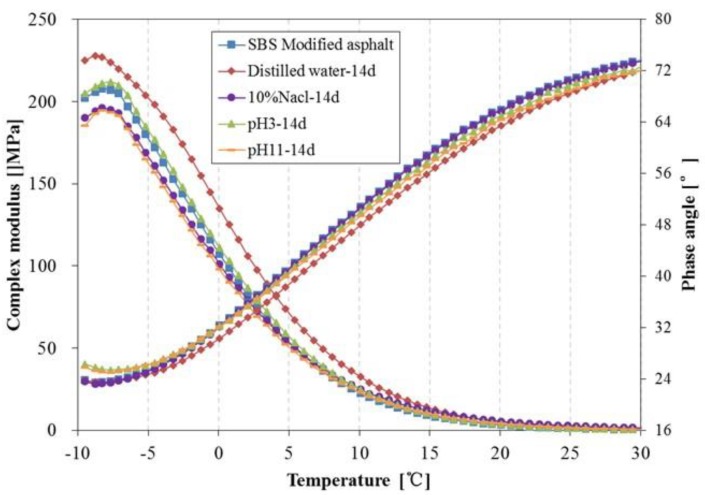
Complex modulus and phase angle of SBS modified asphalt before and after 14 days immersion.

**Figure 15:**
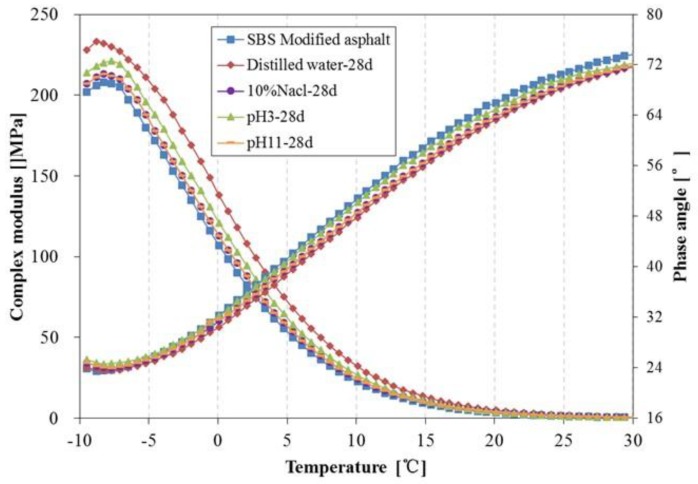
Complex modulus and phase angle of SBS modified asphalt before and after 28 days immersion.

Figure 14 and Figure 15 present the complex modulus and phase angle of SBS modified asphalt after 14 days and 28 days immersion, respectively. From Figure 14 and Figure 15, the effects of immersion in water solutions on the complex modulus of SBS modified asphalt are similar to that of 70^#^ asphalt. However, there are some differences in the effects of the phase angle between SBS modified asphalt and 70^#^ asphalt; the phase angle of SBS modified asphalt after immersion is lower than the original asphalt, and the descent range is lower than that of 70^#^ asphalt. It seems that the modification of bitumen by SBS can lead to less change in the rheological properties when subjected to immersion in water solute. Additionally, among these different water solution immersion conditions, the effect of distilled water on the complex modulus and phase angle is the most remarkable. The asphalt become stiffer after being immersed in distilled water, especially for those samples immersed for 28 days. The results also indicated that to a certain extent, the rheological properties show a corresponding relationship to the change in chemical components. Asphaltene is the main component that affects the rheological property of asphalt [32]. With the prolonged water solute immersion time, the relative amount of asphaltene in the surface of the asphalt increases gradually, which results in the increase in the complex modulus.

**Figure 16:**
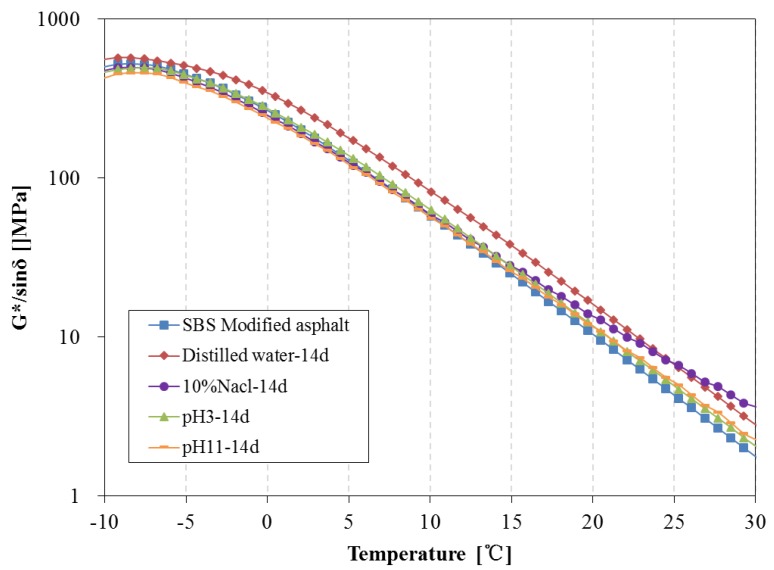
Rutting parameter of SBS modified asphalt before and after 14 days immersion.

**Figure 17:**
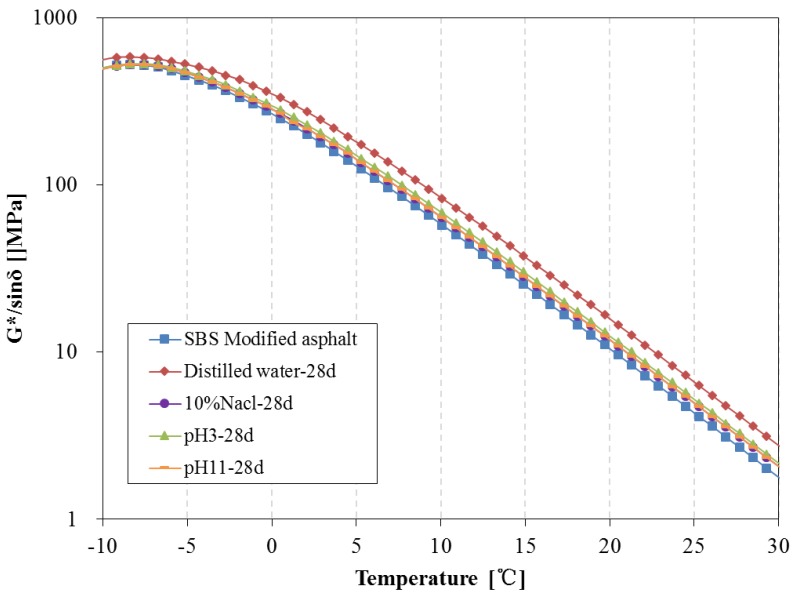
Rutting parameter of SBS modified asphalt before and after 28 days immersion.

Figure 16 and Figure 17 present the rutting parameter of SBS modified asphalt after 14 days and 28 days water solute immersion, respectively. The rutting parameter of asphalt immersed in distilled water is distinctly higher than that of the original asphalt after 14 days, however, the rutting parameter of asphalt immersed in the water solution with the aqueous solute is close to the original asphalt over 14 days and are obviously higher than that of the original asphalt after 28 days.

**Figure 18:**
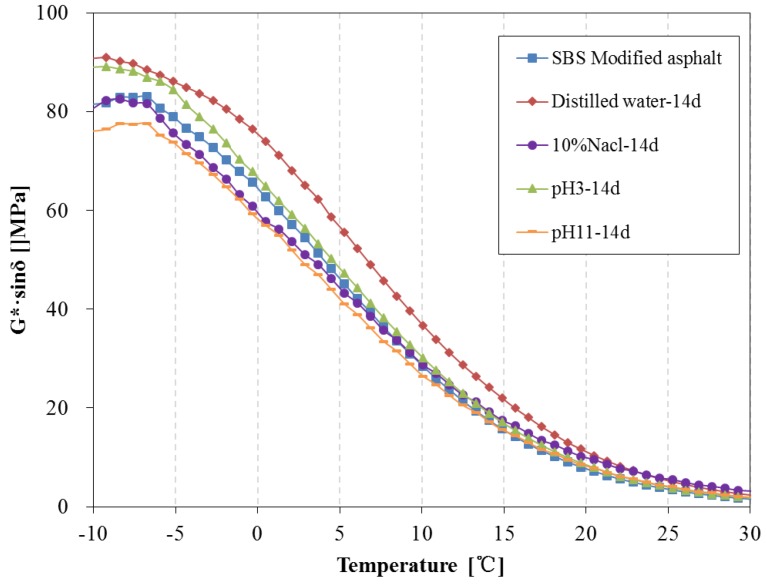
Fatigue factor of SBS modified asphalt before and after 14 days immersion.

**Figure 19:**
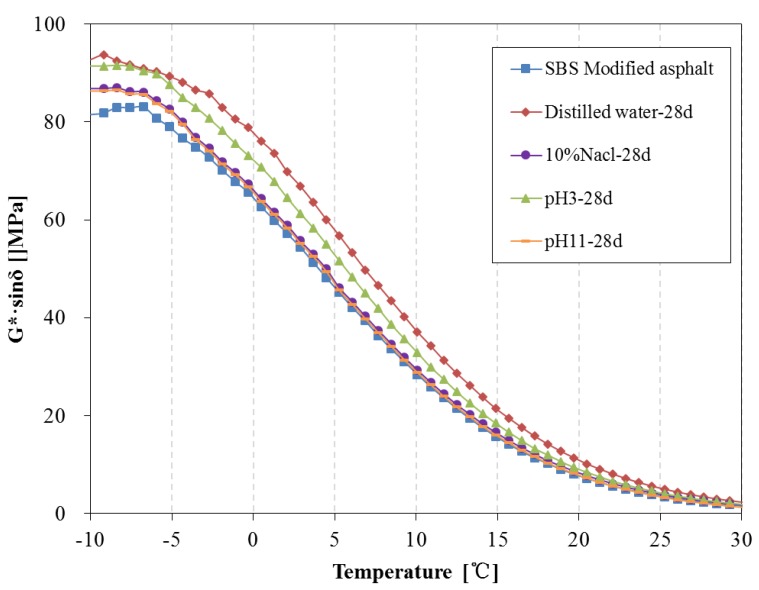
Fatigue factor of SBS modified asphalt before and after 28 days immersion.

Figure 18 and Figure 19 present the G*·sinδ of SBS modified asphalt after 14 days and 28 days water solute immersion, respectively. The fatigue factor of SBS modified asphalt immersed in distilled water and pH3 acidic solution is distinctly higher than that of original SBS modified asphalt over 14 days, however, the fatigue factor of asphalt immersed in pH11 alkaline solution and 10% NaCl saline solution is lower than the original SBS modified asphalt and are slightly higher than that of the original asphalt after 28 days.

All the results above demonstrate that all of these four kinds of water solutions can change the rheological performance of asphalt, the aqueous solute composition plays an important role in the properties of asphalt after water solute exposure, and the sum of these chemical processes leads to asphalt hardening and higher brittleness. The aging process, the dissolving and removal processes, is slow in distilled water, however the process is accelerated with the addition of aqueous solute compositions such as those found in acid rain areas, the seaboard, and in areas with saline and alkaline soil.

## 4. Conclusions

The effects of water solute exposure on asphalt chemistry and rheology were investigated by TLC-FID, FTIR spectra and DSR. Based on the analysis above, the following conclusions can be drawn:

(1) With the water-soluble substances of asphalt solvated and displaced by water, some water-insoluble light component fractions also can seep up and separate from the surface of the asphalt. Moisture damage is associated with dissolution and removes some polar or nonpolar compounds from the asphalt surface to water closely.

(2) Unlike common oxidized aging in heat-oxidized and UV-oxidized aging, saturate fractions may be removed onto the surface of water solutions and form a light-yellow oil patch, that reduces the content of saturates in asphalt after water solute immersion.

(3) Compared with distilled water, the addition of aqueous solute compositions can remarkably affect the chemical properties of asphalt during water solute exposure, and it was clear that the effects could be ordered as follows, pH11 alkaline solution > pH3 acidic solution > 10% NaCl saline solution. It may be that the addition of the aqueous solute compositions accelerates the dissolving and ionizing of some soluble compounds from the asphalt surface to water solutions and degrade the chemical properties of asphalt and the extent of moisture damage.

(4) Immersion in water solutions induced changes in the chemical composition and structure of asphalt. As a result, the newly-formed material structure caused certain effects on the rheological properties of asphalt. While the complex modulus, rutting parameter and fatigue factor increased and the phase angle decreased in distilled water, the adding of aqueous solute brought about the opposite effects in the early phases of immersion. With the immersion time prolonged, the relative amount of asphaltene in the surface of asphalt increased gradually, which resulted in an increase in the complex modulus and a decrease in the phase angle.

The changes in the chemical composition and rheological properties of asphalt binders during immersion in water solutions were investigated in this paper, however, the interactions between water solutions and asphalt and the influence on the micro-domains’ mechanical and conventional physical properties of asphalt binders are still not clear. Investigations of the effects of exposure to water solutions on the micro-domains’ mechanical properties and conventional physical properties such as penetration, softening point and ductility, will be carried out in our future research.

## Figures and Tables

**Table 1:** Basic properties of 70^#^ asphalt and SBS modified asphalt.

Properties	Units	70^#^ Asphalt	SBS Modified Asphalt	Test Specification
Penetration (25 °C, 10 g, 5 s)	0.1 mm	68	56	ASTM D5-61
Softening point	°C	47.2	74.0	ASTM D36-26
Ductility	cm	63.2	68.0	ASTM D113

**Table 2:** Component fractions of 70^#^ asphalt before and after water solute immersion test.

Sample		Saturates (%)	Aromatics (%)	Resins (%)	Asphaltenes (%)
Original sample		15.35	38.88	34.44	11.33
Distilled water	7 days	15.11	38.77	34.15	11.97
	14 days	15.02	37.79	34.68	12.51
10% NaCl salt solution	7 days	15.57	39.04	32.83	12.56
	14 days	15.36	37.56	33.02	14.06
pH3 acid solution	7 days	11.00	39.28	31.78	17.94
	14 days	12.29	38.32	30.49	18.89
pH11 alkali solution	7 days	16.73	39.03	27.70	16.54
	14 days	13.57	38.64	28.11	19.68

**Table 3:** Group indices of 70^#^ asphalt before and after water solute immersion.

Results	Original Sample	Distilled Water		10% NaCl Saline Solution		pH3 Acid Solution		pH11 Alkaline Solution	
		7 days	14 days	7 days	14 days	7 days	14 days	7 days	14 days
Carbonyl index	0.0003	0.0009	0.0012	0.0009	0.0051	0.0106	0.0121	0.0148	0.0151
Sulphoxide index	0.0348	0.0387	0.0412	0.0567	0.0598	0.0651	0.0662	0.0717	0.0778

**Table 4:** Group indices of SBS modified asphalt before and after water solute immersion.

Results	Original Sample	Distilled Water		10% NaCl Saline Solution		pH3 Acid Solution		pH11 Alkaline Solution	
		14 days	28 days	14 days	28 days	14 days	28 days	14 days	28 days
Carbony1 index	0.0108	0.0109	0.0135	0.0110	0.0135	0.0117	0.0201	0.0129	0.0216
Sulphoxide index	0.0198	0.0202	0.0218	0.0220	0.0259	0.0223	0.0257	0.0233	0.0265
Butadiene index	0.0252	0.0245	0.0237	0.0229	0.0229	0.0228	0.0205	0.0220	0.0207
